# Impact of emergency physician-performed ultrasound for the evaluation of patients with acute abdominal pain, prospective randomized dual Centre study: study protocol for a diagnostic trial

**DOI:** 10.1186/s13063-022-06755-2

**Published:** 2022-09-24

**Authors:** François Brau, Stéphanie Martin, Quentin Le Bastard, Patricia Ricaud, Arnaud Legrand, Emmanuel Montassier, Philippe Le Conte

**Affiliations:** 1 Emergency Department, Centre Hospitalier Départemental, La Roche/Yon, France; 2 Clinical Research Department, Centre Hospitalier Départemental, La Roche/Yon, France; 3 Emergency Department, Centre Hospitalier Universitaire, Nantes, France; 4 Faculté de médecine, Nantes Université, Nantes, France; 5 Clinical Research Department, Nantes University Hospital, Nantes, France

**Keywords:** Abdominal pain, Diagnostic, Point-of-care ultrasound, Emergency medicine

## Abstract

**Background:**

Abdominal pain is frequent in patients consulting in emergency departments. The aim of this study is to determine the diagnosis efficacy of point-of-care ultrasound (POCUS) in patients consulting in the ED for acute abdominal pain by comparing the rate of exact diagnostic between the two arms (with or without POCUS), according to the index diagnostic established by an adjudication committee.

**Methods:**

It is a randomized, controlled, open and interventional study in two emergency departments. The included patients will be adults admitted for acute abdominal pain. Exclusion criteria will be a documented end-of-life, an immediate need of life-support therapy and pregnant or breast-feeding women. Patients will be randomized in intervention (POCUS) or control groups. POCUS will only be performed by trained physicians and will be added to the diagnosis procedure in the intervention group. In the control group, the diagnosis will be established after clinical examination and reception of biological analysis results. In the interventional group, the diagnosis will be established after a clinical exam, biological analysis reception and POCUS. An adjudication committee will review all data from case report forms and will determine the index diagnosis which will be used for the analysis.

The primary endpoint will be the comparison of the rate of exact diagnostic between the two arms according to the adjudication committee diagnostic. Secondary endpoints will be the comparison between the two groups for diagnostic delay, duration of ED stay, diagnostic performances for non-specific abdominal pain and hospitalization rate. The primary endpoint will be compared between the two groups using a mixed model taking into account the recruiting centre. Delays will be compared by a mixed linear generalized model. Diagnostic performances will be estimated with their 95% confidence intervals. For a correct diagnostic rate of 57% in the control group and 74% in the intervention group with a 0.05 alpha risk and a 80% power, 244 patients will be required.

**Discussion:**

POCUS diagnostic abilities have been mainly demonstrated in monocentric studies but the level of evidence of its diagnostic efficacy remains controversial in particular in Europe. The aim of this study is to address this question with a rigorous methodology.

**Trial registration:**

ClinicalTrials.gov NCT04912206. Registered on June 3, 2021.

## Introduction

Acute abdominal pain is one of the most frequent complaints of patients presenting to the emergency department (ED) [1]. In the US, in 2007, these patients represented 6.5% of the total ED census [1]. Acute abdominal pain can arise from many causes, including surgical, medical, and also non-specific abdominal pains [2]. Acute abdomen remains a continuing diagnosis challenge for emergency physicians (EP), even if diagnosis performance has improved over the years, especially due to the widespread availability of advanced imaging tools, such as computed tomography (CT) or ultrasound (US) [1, 3]. Currently, evaluating acute abdominal pain relies on the physical examination, laboratory tests, and in many cases, imaging procedures. In the context of limited radiology department resources, this leads to long waiting times in the ED.

Point-of-care ultrasound (POCUS), performed by clinicians at the bedside, is increasingly used in the ED [4]. Recently, it has become an integral part of the EM curriculum [5]. US is particularly suited to assess patients with acute abdominal pain as many organs are easily explored [6]. Lindelius demonstrated that a surgeon-performed US increased diagnostic accuracy in patients with acute abdominal pain [7]. Moreover, POCUS decreased short-term examinations [8] and increased patient’s satisfaction. It was also demonstrated that POCUS could result in fewer further requested examinations, fewer admissions and shorter lead times to surgery, without significant side effects [9]. Another study showed that POCUS increased diagnostic accuracy and the planned diagnostic imaging work-up in a population of 128 ED patients with non-specific abdominal pain [10]. POCUS is usually used as a first-line imaging procedure, followed by a computed tomography if needed. This strategy has been considered as the most accurate according to sensitivity and exposure to radiation [2].

In summary, POCUS diagnostic abilities have been demonstrated, in monocentric studies [2, 7, 8, 10]. However, the level of evidence of POCUS diagnostic efficiency remains controversial. We thus aimed to investigate the efficacy of an early POCUS on diagnostic accuracy by a bicentric, randomized, and controlled study.

## Methods and analysis

### Design

It will be a randomized, controlled, open and interventional study. POCUS will be added on top of the usual diagnosis procedure in the intervention group while it will not be used in the control group. The two recruiting centres are a university hospital and a more rural community hospital, this pragmatic approach intends to validate the added diagnostic value of POCUS in various settings.

### Objectives

To determine the diagnostic efficacy of POCUS in patients consulting in the ED for acute abdominal pain by comparing the rate of exact diagnostic between the two arms (with or without POCUS), according to the index diagnostic established by an adjudication committee. The diagnosis will be the one given by the physician before he has any access to the results of complementary examination performed in radiology.

### Participants

The included patients will be adults admitted for acute abdominal pain.

#### Inclusion criteria

Patients older than 18 years

Admitted in the ED or Nantes University Hospital or La Roche/Yon Hospital

Admitted for abdominal pain lasting for less than 5 days

Presence in the ED at that time of an EP trained in POCUS

#### Exclusion criteria

Documented end-of-life with a do-not-resuscitate order

Immediate need of life-support therapy

Patient sent to the ED by an out-of-hospital practitioner

Unable to speak and understand French

Pregnant and breast-feeding women

Patients non-affiliated to social security

Patient under guardianship

#### Drop out criteria

Patient’s death

Withdrawal of consent

### Intervention

In the control group, the diagnosis will be established after clinical examination and reception of biological analysis results. In the interventional group, a POCUS performed in the ED by a local investigator will be added and the diagnosis will be established after a clinical exam, biological analysis reception and POCUS. POCUS will only be performed by physicians who have completed a validated training program.

Furthermore, before study initiation, refresh sessions focused on acquisition techniques and pathological findings will be organized in the two participating ED. They will include hands-on sessions and presentations of pathological loops and images. Such refresher courses have been efficient to increase the overall confidence of operators [11].

### Description of POCUS

POCUS, performed in B mode only with a curvilinear probe, will assess the major spots and search for main anomalies (Table 1). It will be performed using Mindray® ME8 or TE7 or Philips® CX50 on a patient in a lying position. The following spots will be explored:

- Epigastric sagittal view to explore the aorta.
- Oblique view of the right lateral wall to explore the gallbladder, the Morrison pouch and the right kidney.
- Oblique view of the left lateral wall to explore the Kohler pouch and the left kidney.
- Hypogastric transversal and sagittal to explore the bladder, the Douglas pouch and the ovaries in women.
- Transversal view of the right lower quadrant in search of the appendix.
- Transversal exploration of the anterior abdominal wall in search of dilated bowel loops.

**Table 1:** Spots and focussed anomalies visualized by POCUS in echoPAIN study

Organ	Pathologic finding (illness)
Abdominal aorta	Aneurysm (dilated aorta) Aortic dissection (presence of a flap)
Gallbladder	Cholelithiasis (presence of lithiasis) cholecystitis (Murphy sign, wall thickening and presence of lithiasis)
Kidneys	Renal colic (hydronephrosis)
Bladder	Dilated bladder
Peritoneum (cul-de-sac)	Presence of free fluid in the peritoneal pouches
Small bowel loops	Bowel obstruction (dilated, incompressible loops with back-and-forth liquid movements)
Appendix	Appendicitis (non-compressible appendix with diameter > 6 mm)
Ovaries	Ovarian cysts or mass

### Adjudication committee

An adjudication committee will be established, involving three independent experts in emergency medicine, radiology, and abdominal surgery. They will review all data from case report forms including routine diagnosis procedures (clinical, radiological and laboratory tests) and will determine the index diagnosis which will be used for the analysis.

### Endpoints

#### Primary endpoint

- Comparison of the rate of exact diagnostic after clinical exam and biological results with POCUS (interventional arm) or without (control arm) according to the adjudication committee diagnostic.

#### Secondary endpoints

- Comparison between the two groups:
  - Delay between admission and diagnostic
  - Duration of ED stay
  - Diagnostic performances for non-specific abdominal pain
  - Prescription of biological and radiological exams during the ED stay
  - Hospitalization rate
  - Rehospitalization rate
- In the interventional group (with POCUS):
  - Diagnostic concordance between POCUS and radiological US if performed
  - Contribution of POCUS to the diagnostic
  - Duration and self-assessed difficulty of POCUS

### Randomization

Patients will be randomized 1:1 to POCUS or control group by a computed-based program in random block sizes and stratified by centres.

### Recruiting centres

The recruiting centres will be the ED of Nantes University hospital and of La Roche/Yon hospital.

### Allocation concealment mechanism

POCUS results will be concealed in the files transmitted to the adjudication committee

### Implementation

The implementation is scheduled as follows:

- Recruitment of emergency physicians with validated ultrasound skills (national training program)
- Information of the registered nurses and other emergency physicians on the onset of the study
- Implementation of the whole documents and of the patient’s pathways in the two ED by the local research teams

### Monitoring

A monitoring process will be performed in the two sites in order to control the validity of data. An audit by the public health authorities could be done.

### Amendments

According to the French law, possible amendments would be submitted to the ethics committee before being transcribed in a new version of the protocol.

### Sample size calculation

According to Lindelius [7], a correct diagnosis has been reached in 56.8% of patients consulting to the ED for acute abdominal pain after the clinical exam and biological results. The hypothesis is based on an improvement of 30% of diagnostic accuracy by POCUS. Jang [10] demonstrated an improvement of diagnostic accuracy by 45% while Lindelius by 8% [7, 8]. A correct diagnostic rate of 57% is expected in the control group and 74% in the intervention group. With a 0.05 alpha risk and a 80% power, 244 patients will be required. A 5% attrition rate (patients randomized but presenting exclusion criteria) is expected; thus, 256 randomized patients are needed for this study.

### Data management

eCRFs will be used via a web-based interface. All data will be stored in Nantes University Hospital’s secured databases. The data management team will be responsible for the entire process. Data will be anonymized with an incremental number assigned to each patient. The final database will be available only to the adjudication committee.

### Monitoring

Monitoring will be performed both by electronic surveillance of recruitment and data quality. It will be done by the Clinical Research Department of Nantes University Hospital.

### Statistical analysis

Baseline characteristics for each group will be descriptively presented using frequencies and percentages for categorical variables and means and SDs (or median and IQR if appropriate) for continuous variables. An intermediate analysis will not be performed. The primary endpoint (rate of exact diagnostic) will be compared between the two groups (with or without POCUS) using a mixed model taking into account the recruiting centre (factor of stratification of randomization). The delays will be compared by a mixed linear generalized model adjusted on the recruiting centres for the same reason as for the primary endpoint. Sensitivity, specificity and positive and negative predictive values will be estimated with their 95% confidence intervals. The number of biological exams will be compared between the two groups using a Poisson model. The rates of readmission and hospitalization will be compared using a logistic generalized mixed model adjusted on the centre.

In the interventional group (with POCUS), the concordance between the POCUS and ultrasound performed by a radiologist will be assessed by Kappa coefficient. Duration and self-assessed difficulty of POCUS will be described by means, SDs, medians, minimums and maximums.

## Discussion

Abdominal POCUS performed by EP might improve the overall diagnostic procedure in patients consulting to the ED for abdominal pain. Some studies have demonstrated its ability to increase the diagnostic accuracy while decreasing the number of short-term complementary examinations. However, there is still a need for a confirmation since the published studies were mostly monocentric, performed by surgeons or EP and did not all report the abnormalities which could be seen. EchoPAIN has the potential to provide a strong response to this question since it is a randomized control trial involving EP with similar POCUS backgrounds.

## Trial status

The current protocol version is V2 from March 2021, 21, the recruitment began on July 2021, 23 and the expected completion date is June 2022.

## Data Availability

Raw data will be available on reasonable request.

## Abbreviations

POCUS: Point-of-care ultrasound
ED: Emergency department
EP: Emergency physicians
CT: Computed tomography
US: Ultrasound
EM: Emergency medicine
ECRFs: Electronic case report files
